# Tetra­ethyl­ammonium bicarbonate trihydrate

**DOI:** 10.1107/S1600536811026080

**Published:** 2011-07-09

**Authors:** Heping Li, Yimin Hou, Yunxia Yang

**Affiliations:** a Henan University of Traditional Chinese Medicine, Zhengzhou 450008, People’s Republic of China; b Key Laboratory of Polymer Materials of Gansu Province, Ministry of Education, College of Chemistry and Chemical Engineering, Northwest Normal University, Lanzhou 730070, Gansu, People’s Republic of China

## Abstract

In the title compound, C_8_H_20_N^+^·CHO_3_^−^·3H_2_O, the bicarbon­ate anion, which has a small mean deviation from the plane of 0.0014 Å, fully utilises its three O and one H atom to form various O—H⋯O hydrogen bonds with the three water mol­ecules in the asymmetric unit, generating a hydrogen-bonded layer, which extends along (10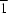). The tetra­ethyl­ammonium cations, as the guest species, are accommodated between every two neighboring layers, constructing a sandwich-like structure with an inter­layer distance of 7.28 Å.

## Related literature

For the crystal structure of tetra­ethyl­ammonium bicarbonate monohydrate clathrate, see: Li *et al.* (2003 ▶). For O—H⋯O hydrogen bonds, see: Steiner (2002 ▶). For polymorphism see Kumar *et al.* (2002 ▶). 
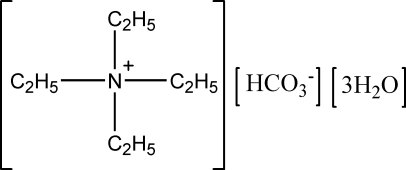


## Experimental

### 

#### Crystal data

C_8_H_20_N^+^·CHO_3_^−^·3H_2_O
*M* *_r_* = 245.32
Monoclinic, 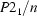

*a* = 7.6633 (1) Å
*b* = 12.9627 (3) Å
*c* = 14.2683 (3) Å
β = 99.932 (1)°
*V* = 1396.13 (5) Å^3^
*Z* = 4
Mo *K*α radiation
μ = 0.10 mm^−1^
*T* = 296 K
0.61 × 0.29 × 0.18 mm

#### Data collection

Bruker SMART APEX diffractometer
Absorption correction: multi-scan (*SADABS*; Sheldrick, 1996 ▶) *T*_min_ = 0.854, *T*_max_ = 1.000
8465 measured reflections
3480 independent reflections
2466 reflections with *I* > 2σ(*I*)
*R*_int_ = 0.018

#### Refinement

*R*[*F* ^2^ > 2σ(*F* ^2^)] = 0.045
*wR*(*F* ^2^) = 0.138
*S* = 1.02
3480 reflections
166 parameters
10 restraints
H atoms treated by a mixture of independent and constrained refinement
Δρ_max_ = 0.16 e Å^−3^
Δρ_min_ = −0.17 e Å^−3^

### 

Data collection: *APEX2* (Bruker, 2009 ▶); cell refinement: *SAINT* (Bruker, 2009 ▶); data reduction: *SAINT*; program(s) used to solve structure: *SHELXS97* (Sheldrick, 2008 ▶); program(s) used to refine structure: *SHELXL97* (Sheldrick, 2008 ▶); molecular graphics: *SHELXTL* (Sheldrick, 2008 ▶); software used to prepare material for publication: *SHELXL97* and *publCIF* (Westrip, 2010 ▶).

## Supplementary Material

Crystal structure: contains datablock(s) I, global. DOI: 10.1107/S1600536811026080/nr2008sup1.cif

Structure factors: contains datablock(s) I. DOI: 10.1107/S1600536811026080/nr2008Isup2.hkl

Additional supplementary materials:  crystallographic information; 3D view; checkCIF report

## Figures and Tables

**Table 1:** Hydrogen-bond geometry (Å, °)

*D*—H⋯*A*	*D*—H	H⋯*A*	*D*⋯*A*	*D*—H⋯*A*
O1*W*—H1*WA*⋯O2^i^	0.83 (1)	2.00 (1)	2.8239 (16)	177 (2)
O1*W*—H1*WB*⋯O3*W*^ii^	0.82 (1)	2.05 (1)	2.8666 (19)	173 (2)
O2*W*—H2*WA*⋯O1	0.83 (1)	1.97 (1)	2.7980 (15)	172 (2)
O2*W*—H2*WB*⋯O1*W*^iii^	0.82 (1)	2.01 (1)	2.8229 (16)	171 (2)
O3—H3⋯O1^iv^	0.83 (1)	1.85 (1)	2.6676 (15)	172 (2)
O3*W*—H3*WA*⋯O2	0.83 (1)	2.06 (1)	2.8422 (18)	157 (2)
O3*W*—H3*WB*⋯O2*W*	0.81 (1)	2.07 (2)	2.8099 (19)	152 (3)

Symmetry codes: (i) 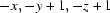; (ii) 
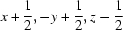
; (iii) 
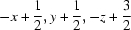
; (iv) 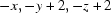.

**Fig. 1.**
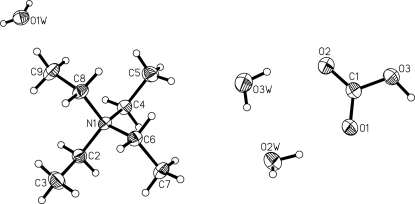
Thermal ellipsoid plot of the title compound at the 30% probability level; hydrogen atoms are drawn as spheres of arbitrary radius.

**Fig. 2.**
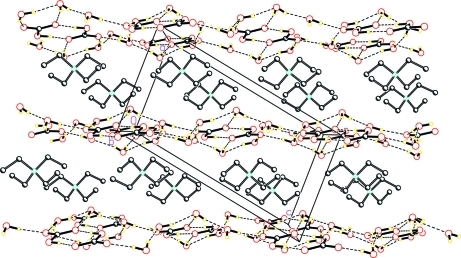
Packing diagram of the title compound; all hydrogen atoms bonded to carbon are omitted for clarity and the cations are represented with the open bonds.

**Fig. 3.**
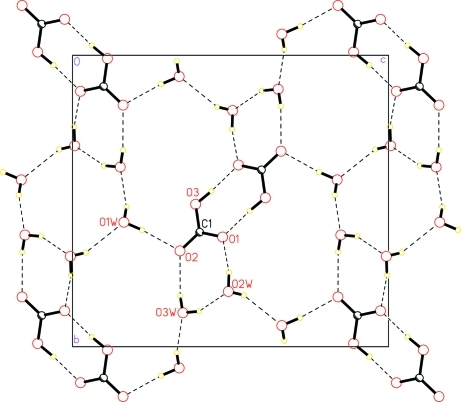
Hydrogen-bonded linking pattern of the host layer in the crystal structure of the title compound.

## supplementary crystallographic information

### Comment

Polymorphism is the existence of the same chemical substance in at least two different crystalline arrangements of molecules (Kumar *et al.* 2002). It is helpful to understand polymorphism to explore different crystal structures which are not qualified polymorphs but are also constructed with the same components. In 2003, the crystal structure of tetraethylammonium bicarbonate monohydrate clathrate (C_8_H_20_N^+^.CHO_3_^-^.H_2_O, 1) has been reported (Li *et al.* 2003). Here we reported the crystal structure of tetraethylammonium bicarbonate trihydrate clathrate (C_8_H_20_N^+^.CHO_3_^-^.3H_2_O, 2), in which the same components were used to obtain the crystal but the difference of the amount of water molecules in the asymmetric unit results in the final different packing model compared with compound 1. In addition, it should be noted that, in our experiment, 4,4'-oxybis(benzoic acid) was used to be the host molecule to obtain the acid-base inclusion compound, but after the data collection and determination, it was found that bicarbonate anion, which was finally determined according to the corresponding C—O bond lengths and O—C—O angles existed in the similar crystal structure of compound 1, take the place of the acid to interact with the related base to generate compound 2. In compound 1, one bicarbonate anion and one water molecule interacting with each other through O—H···O hydrogen bonds constitute a zigzag ribbon and are arranged in un-closed channels generated from tetraethylammonium cations. Comparatively, one bicarbonate anion and three water molecules in compound 2 form more O—H···O hydrogen bonds to construct the hydrogen-bonded layer and tetraethylammonium cations are contained between the layers to display the typical sandwich-like structure. Obviously, the amount of water molecules has significant effect on constructing different crystal structure between compound 1 and2. Noticeably, in compound 2, the strongest O—H···O hydrogen bond is between the centro-symmetric related bicarbonate anions (the distance of O···O is 2.6654 (16) Å) and other weaker O—H···O contacts involve the participation of water molecules (the corresponding values are from 2.7991 (16) Å to 2.868 (2) Å), which can be compared with the related O···O intervals of compound 1 (O···O distances are 2.619 Å and 2.868 Å) and the corresponding values (2.68 Å ~ 3.11 Å) of the reference (Steiner, 2002).

### Experimental

4,4'-Oxybis(benzoic acid) (0.25 mmol, 0.065 g) was dissolved in a water-ethanol (50 ml/100 ml *v*/*v*) mixture. Tetraethylammonium hydroxide (25% aqueous solution) was added to neutralize the acid. The mixture was stirred for about 2 h and set aside to crystallize. Unexpectedly, the crystals involved 4,4'-oxybis(benzoic acid) were not obtained. Instead, colorless block crystals of the title compound were separated after several weeks.

### Refinement

Carbon-bound H-atoms were placed in calculated positions (C—H: 0.96 Å for CH_3_ group and 0.97 Å for CH_2_ group) and were included in the refinement in the riding model approximation, with *U*(H) set to 1.2*Ueq*(C) for CH_2_ group and 1.5*Ueq*(C) for CH_3_ group. The anion and water H-atoms were located in a difference Fourier map, and were refined with a distance restraint of O—H 0.82±0.01 Å and with *U*(H) set to 1.5*Ueq*(O). Meanwhile, for water molecules, H—H distances were also restrained within 1.41±0.02 Å to meet the needs of H—O—H angles.

### Crystal data

**Table:**

C_8_H_20_N^+^·CHO_3_^−^·3H_2_O	*F*(000) = 544
*M**_r_* = 245.32	*D*_x_ = 1.167 Mg m^−^^3^
Monoclinic, *P*2_1_/*n*	Mo *K*α radiation, λ = 0.71073 Å
Hall symbol: -P 2yn	Cell parameters from 2627 reflections
*a* = 7.6633 (1) Å	θ = 3.1–26.7°
*b* = 12.9627 (3) Å	µ = 0.10 mm^−^^1^
*c* = 14.2683 (3) Å	*T* = 296 K
β = 99.932 (1)°	Block, colourless
*V* = 1396.13 (5) Å^3^	0.61 × 0.29 × 0.18 mm
*Z* = 4	

### Data collection

**Table:**

Bruker SMART APEX diffractometer	3480 independent reflections
Radiation source: fine-focus sealed tube	2466 reflections with *I* > 2σ(*I*)
graphite	*R*_int_ = 0.018
phi and ω scans	θ_max_ = 28.5°, θ_min_ = 2.1°
Absorption correction: multi-scan (*SADABS*; Sheldrick, 1996)	*h* = −10→8
*T*_min_ = 0.854, *T*_max_ = 1.000	*k* = −17→15
8465 measured reflections	*l* = −12→19

### Refinement

**Table:**

Refinement on *F*^2^	Primary atom site location: structure-invariant direct methods
Least-squares matrix: full	Secondary atom site location: difference Fourier map
*R*[*F*^2^ > 2σ(*F*^2^)] = 0.045	Hydrogen site location: inferred from neighbouring sites
*wR*(*F*^2^) = 0.138	H atoms treated by a mixture of independent and constrained refinement
*S* = 1.02	*w* = 1/[σ^2^(*F*_o_^2^) + (0.0697*P*)^2^ + 0.1916*P*] where *P* = (*F*_o_^2^ + 2*F*_c_^2^)/3
3480 reflections	(Δ/σ)_max_ = 0.001
166 parameters	Δρ_max_ = 0.16 e Å^−^^3^
10 restraints	Δρ_min_ = −0.17 e Å^−^^3^

### Special details

**Table:**

Geometry. All e.s.d.'s (except the e.s.d. in the dihedral angle between two l.s. planes) are estimated using the full covariance matrix. The cell e.s.d.'s are taken into account individually in the estimation of e.s.d.'s in distances, angles and torsion angles; correlations between e.s.d.'s in cell parameters are only used when they are defined by crystal symmetry. An approximate (isotropic) treatment of cell e.s.d.'s is used for estimating e.s.d.'s involving l.s. planes.
Refinement. Refinement of *F*^2^ against ALL reflections. The weighted *R*-factor *wR* and goodness of fit *S* are based on *F*^2^, conventional *R*-factors *R* are based on *F*, with *F* set to zero for negative *F*^2^. The threshold expression of *F*^2^ > σ(*F*^2^) is used only for calculating *R*-factors(gt) *etc*. and is not relevant to the choice of reflections for refinement. *R*-factors based on *F*^2^ are statistically about twice as large as those based on *F*, and *R*- factors based on ALL data will be even larger.

### Fractional atomic coordinates and isotropic or equivalent isotropic displacement parameters (Å^2^)

**Table:**

	*x*	*y*	*z*	*U*_iso_*/*U*_eq_	
N1	0.23247 (12)	0.27367 (8)	0.83104 (6)	0.0337 (2)	
O1	0.02644 (15)	0.87654 (8)	0.97489 (8)	0.0608 (3)	
O1W	0.24867 (16)	0.07251 (10)	0.33750 (8)	0.0642 (3)	
H1WA	0.218 (3)	0.0994 (16)	0.2845 (10)	0.096*	
H1WB	0.308 (3)	0.0199 (12)	0.3364 (16)	0.096*	
C1	−0.08821 (18)	0.89307 (11)	0.90217 (9)	0.0453 (3)	
O2	−0.14805 (14)	0.82902 (9)	0.84014 (8)	0.0594 (3)	
O2W	0.21273 (15)	0.68993 (10)	0.99356 (9)	0.0641 (3)	
H2WA	0.165 (3)	0.7475 (10)	0.9923 (15)	0.096*	
H2WB	0.212 (3)	0.6570 (14)	1.0426 (11)	0.096*	
C2	0.40040 (16)	0.21074 (11)	0.86043 (9)	0.0443 (3)	
H2A	0.4158	0.1663	0.8077	0.053*	
H2B	0.5004	0.2577	0.8714	0.053*	
O3	−0.15534 (16)	0.98874 (8)	0.88939 (8)	0.0632 (3)	
H3	−0.109 (3)	1.0260 (15)	0.9338 (12)	0.095*	
O3W	−0.05481 (19)	0.61659 (11)	0.84914 (10)	0.0767 (4)	
H3WA	−0.110 (3)	0.6718 (13)	0.8393 (18)	0.115*	
H3WB	0.017 (3)	0.6182 (19)	0.8983 (12)	0.115*	
C3	0.4058 (3)	0.14500 (13)	0.94748 (12)	0.0654 (4)	
H3A	0.5164	0.1086	0.9602	0.098*	
H3B	0.3099	0.0964	0.9370	0.098*	
H3C	0.3945	0.1880	1.0009	0.098*	
C4	0.26289 (17)	0.33841 (11)	0.74702 (9)	0.0428 (3)	
H4A	0.3656	0.3819	0.7671	0.051*	
H4B	0.2907	0.2926	0.6979	0.051*	
C5	0.1098 (2)	0.40604 (13)	0.70431 (11)	0.0599 (4)	
H5A	0.1407	0.4439	0.6517	0.090*	
H5B	0.0832	0.4535	0.7516	0.090*	
H5C	0.0079	0.3639	0.6825	0.090*	
C6	0.19457 (17)	0.34029 (10)	0.91255 (9)	0.0418 (3)	
H6A	0.0877	0.3797	0.8907	0.050*	
H6B	0.1710	0.2954	0.9633	0.050*	
C7	0.3400 (2)	0.41413 (12)	0.95290 (11)	0.0542 (4)	
H7A	0.3047	0.4526	1.0040	0.081*	
H7B	0.3621	0.4607	0.9040	0.081*	
H7C	0.4460	0.3761	0.9765	0.081*	
C8	0.07102 (17)	0.20495 (11)	0.80381 (10)	0.0460 (3)	
H8A	0.0591	0.1620	0.8580	0.055*	
H8B	−0.0334	0.2484	0.7905	0.055*	
C9	0.0748 (2)	0.13609 (13)	0.71913 (13)	0.0655 (4)	
H9A	−0.0319	0.0958	0.7070	0.098*	
H9B	0.1754	0.0909	0.7321	0.098*	
H9C	0.0834	0.1776	0.6644	0.098*	

### Atomic displacement parameters (Å^2^)

**Table:**

	*U*^11^	*U*^22^	*U*^33^	*U*^12^	*U*^13^	*U*^23^
N1	0.0328 (5)	0.0355 (5)	0.0333 (5)	−0.0040 (4)	0.0068 (4)	−0.0037 (4)
O1	0.0708 (7)	0.0493 (6)	0.0566 (6)	0.0134 (5)	−0.0049 (5)	0.0020 (5)
O1W	0.0656 (7)	0.0716 (8)	0.0541 (6)	−0.0014 (6)	0.0067 (5)	−0.0011 (6)
C1	0.0479 (7)	0.0450 (8)	0.0446 (7)	0.0014 (6)	0.0123 (6)	0.0060 (6)
O2	0.0651 (6)	0.0528 (6)	0.0577 (6)	0.0023 (5)	0.0035 (5)	−0.0053 (5)
O2W	0.0592 (6)	0.0610 (7)	0.0716 (7)	0.0131 (5)	0.0106 (5)	0.0068 (6)
C2	0.0401 (6)	0.0455 (8)	0.0464 (7)	0.0048 (5)	0.0052 (5)	−0.0046 (6)
O3	0.0769 (8)	0.0459 (6)	0.0593 (7)	0.0090 (5)	−0.0094 (5)	0.0057 (5)
O3W	0.0932 (10)	0.0618 (8)	0.0705 (8)	0.0027 (7)	0.0013 (7)	−0.0066 (6)
C3	0.0826 (11)	0.0567 (10)	0.0540 (9)	0.0176 (8)	0.0034 (8)	0.0062 (7)
C4	0.0467 (7)	0.0460 (7)	0.0373 (6)	−0.0067 (6)	0.0116 (5)	0.0010 (5)
C5	0.0662 (9)	0.0568 (9)	0.0532 (8)	−0.0010 (7)	0.0005 (7)	0.0136 (7)
C6	0.0430 (6)	0.0458 (7)	0.0383 (6)	0.0007 (5)	0.0117 (5)	−0.0073 (5)
C7	0.0627 (8)	0.0503 (8)	0.0483 (8)	−0.0063 (7)	0.0054 (6)	−0.0153 (6)
C8	0.0411 (6)	0.0475 (8)	0.0488 (7)	−0.0141 (6)	0.0060 (5)	−0.0028 (6)
C9	0.0682 (10)	0.0556 (10)	0.0685 (10)	−0.0155 (8)	−0.0001 (8)	−0.0204 (8)

### Geometric parameters (Å, °)

**Table:**

N1—C4	1.5141 (15)	C3—H3C	0.9600
N1—C6	1.5162 (15)	C4—C5	1.507 (2)
N1—C8	1.5197 (15)	C4—H4A	0.9700
N1—C2	1.5203 (16)	C4—H4B	0.9700
O1—C1	1.2569 (17)	C5—H5A	0.9600
O1W—H1WA	0.829 (9)	C5—H5B	0.9600
O1W—H1WB	0.823 (9)	C5—H5C	0.9600
C1—O2	1.2422 (17)	C6—C7	1.5064 (19)
C1—O3	1.3429 (18)	C6—H6A	0.9700
O2W—H2WA	0.830 (9)	C6—H6B	0.9700
O2W—H2WB	0.820 (9)	C7—H7A	0.9600
C2—C3	1.501 (2)	C7—H7B	0.9600
C2—H2A	0.9700	C7—H7C	0.9600
C2—H2B	0.9700	C8—C9	1.506 (2)
O3—H3	0.827 (10)	C8—H8A	0.9700
O3W—H3WA	0.831 (9)	C8—H8B	0.9700
O3W—H3WB	0.812 (9)	C9—H9A	0.9600
C3—H3A	0.9600	C9—H9B	0.9600
C3—H3B	0.9600	C9—H9C	0.9600
			
C4—N1—C6	111.57 (10)	C4—C5—H5A	109.5
C4—N1—C8	110.62 (9)	C4—C5—H5B	109.5
C6—N1—C8	105.95 (9)	H5A—C5—H5B	109.5
C4—N1—C2	106.09 (9)	C4—C5—H5C	109.5
C6—N1—C2	111.05 (9)	H5A—C5—H5C	109.5
C8—N1—C2	111.66 (10)	H5B—C5—H5C	109.5
H1WA—O1W—H1WB	113.7 (19)	C7—C6—N1	115.34 (10)
O2—C1—O1	126.42 (14)	C7—C6—H6A	108.4
O2—C1—O3	115.77 (12)	N1—C6—H6A	108.4
O1—C1—O3	117.81 (13)	C7—C6—H6B	108.4
H2WA—O2W—H2WB	114.8 (19)	N1—C6—H6B	108.4
C3—C2—N1	115.72 (12)	H6A—C6—H6B	107.5
C3—C2—H2A	108.4	C6—C7—H7A	109.5
N1—C2—H2A	108.4	C6—C7—H7B	109.5
C3—C2—H2B	108.4	H7A—C7—H7B	109.5
N1—C2—H2B	108.4	C6—C7—H7C	109.5
H2A—C2—H2B	107.4	H7A—C7—H7C	109.5
C1—O3—H3	109.4 (16)	H7B—C7—H7C	109.5
H3WA—O3W—H3WB	112 (2)	C9—C8—N1	115.14 (11)
C2—C3—H3A	109.5	C9—C8—H8A	108.5
C2—C3—H3B	109.5	N1—C8—H8A	108.5
H3A—C3—H3B	109.5	C9—C8—H8B	108.5
C2—C3—H3C	109.5	N1—C8—H8B	108.5
H3A—C3—H3C	109.5	H8A—C8—H8B	107.5
H3B—C3—H3C	109.5	C8—C9—H9A	109.5
C5—C4—N1	115.30 (11)	C8—C9—H9B	109.5
C5—C4—H4A	108.4	H9A—C9—H9B	109.5
N1—C4—H4A	108.4	C8—C9—H9C	109.5
C5—C4—H4B	108.4	H9A—C9—H9C	109.5
N1—C4—H4B	108.4	H9B—C9—H9C	109.5
H4A—C4—H4B	107.5		

### Hydrogen-bond geometry (Å, °)

**Table:**

*D*—H···*A*	*D*—H	H···*A*	*D*···*A*	*D*—H···*A*
O1W—H1WA···O2^i^	0.83 (1)	2.00 (1)	2.8239 (16)	177 (2)
O1W—H1WB···O3W^ii^	0.82 (1)	2.05 (1)	2.8666 (19)	173 (2)
O2W—H2WA···O1	0.83 (1)	1.97 (1)	2.7980 (15)	172 (2)
O2W—H2WB···O1W^iii^	0.82 (1)	2.01 (1)	2.8229 (16)	171 (2)
O3—H3···O1^iv^	0.83 (1)	1.85 (1)	2.6676 (15)	172 (2)
O3W—H3WA···O2	0.83 (1)	2.06 (1)	2.8422 (18)	157 (2)
O3W—H3WB···O2W	0.81 (1)	2.07 (2)	2.8099 (19)	152 (3)

Symmetry codes: (i) −*x*, −*y*+1, −*z*+1; (ii) *x*+1/2, −*y*+1/2, *z*−1/2; (iii) −*x*+1/2, *y*+1/2, −*z*+3/2; (iv) −*x*, −*y*+2, −*z*+2.
